# Nitrate of Silver in the Vomiting of Pregnancy

**Published:** 1859-09

**Authors:** 


### *Nitrate of Silver in the Vomiting of Pregnancy.*

Dr. Channing states that he has found the administration of nitrate of silver (arg. nit. gr. iv., opii. gr. viii., in pil. xvi, cap. 1, ter) very useful in the vomiting of pregnancy. Writting from Boston on the "moral state" of Massachusets, he represents criminal abortion as a daily practice there, performed in the most unblushing manner, and with complete impunity.

